# Clinical characteristics and outcomes in a cohort of oncologic patients with COVID‐19 during the first year of the pandemic in Mexico

**DOI:** 10.1002/cam4.4582

**Published:** 2022-02-14

**Authors:** Daniel De‐la‐Rosa‐Martinez, Mercedes Aranda‐Audelo, Alexandra Martin‐Onraet, Beda Islas‐Muñoz, Carolina Perez‐Jimenez, Pamela Alatorre‐Fernandez, Patricia Cornejo‐Juarez, Erika Ruiz‐Garcia, Paola Zinser‐Peniche, Luis Nuñez‐Luna, Abelardo Meneses‐Garcia, Angel Herrera‐Gomez, Diana Vilar‐Compte

**Affiliations:** ^1^ Department of Infectious Diseases Instituto Nacional de Cancerologia Mexico City Mexico; ^2^ Plan de Estudios Combinados en Medicina (PECEM), Faculty of Medicine Universidad Nacional Autonoma de Mexico Mexico City Mexico; ^3^ Translational Medicine Laboratory and Department of Gastrointestinal Tumors Instituto Nacional de Cancerologia Mexico City Mexico; ^4^ Division of Pathology Instituto Nacional de Cancerologia Mexico City Mexico; ^5^ Division of Surgical Oncology Instituto Nacional de Cancerologia Mexico City Mexico; ^6^ Present address: Department of Infectious Diseases Hospital General Dr. Manuel Gea Gonzalez Mexico City Mexico

**Keywords:** cancer, COVID‐19, hospitalization, mechanical ventilation, mortality, outcomes, SARS‐CoV‐2

## Abstract

**Background:**

Literature on severe acute respiratory syndrome coronavirus 2 (SARS‐CoV‐2) infection in cancer patients is scarce in Latin America. This population seems to have a higher risk for adverse outcomes. This study aims to correlate clinical characteristics with outcomes in patients with cancer.

**Methods:**

We included all patients with cancer and confirmed SARS‐CoV‐2 infection from April 19 to December 31, 2020, at the Instituto Nacional de Cancerologia, Mexico. Clinical information was obtained from medical and epidemiological records. For the association between variables and hospitalization, invasive mechanical ventilation (IMV), and mortality, univariate and multivariate logistic regression were performed; odds ratios and 95% confidence intervals were calculated.

**Results:**

Four hundred thirty‐three patients were included; 268 (62%) were female, the median age was 55 years. One hundred thirty‐five (31%), 131 (30%), and 93 (21%) patients had obesity, hypertension, and diabetes mellitus (DM), respectively. Three hundred forty‐one (79%) had solid cancer. One hundred seventy (39%) had advanced cancer. Two hundred (46%) patients were hospitalized. Age (*p* < 0.01), male gender (*p* = 0.03), hematological malignancies (HM) (*p* = 0.04) and advanced cancer (*p* = 0.03) increased the risk for hospital admission. Forty‐five (10%) patients required IMV. Age (*p* = 0.02); DM (*p* = 0.04); high C‐reactive protein (*p* < 0.01), and lactate dehydrogenase (*p* = 0.03) were associated with IMV. Mortality within 30 days after diagnosis was 18% (76 cases). Associated characteristics were age (*p* = 0.04) and low albumin (*p* < 0.01).

**Conclusions:**

In this study, patients with cancer showed higher mortality, need for hospitalization, and IMV compared with other non‐cancer cohorts. We did not find an increased risk in mortality for HM. Although our cohort was younger than others previously reported, age was a strong predictor of adverse outcomes. Variables associated with IMV and death were similar to those previously described in cancer patients with COVID‐19.

## Graphical abstract

In this study, we found that patients with cancer had higher mortality, need of hospitalization, and mechanical ventilation compared with other non‐cancer cohorts. Age was the most important risk factor for hospital admission, invasive mechanical ventilation, and death regardless of the type of cancer.

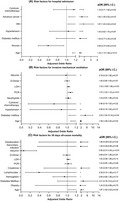

## 1 INTRODUCTION

Coronavirus disease (COVID‐19) is a respiratory tract infection caused by the severe acute respiratory syndrome coronavirus 2 (SARS‐CoV‐2) that has transformed the delivery of healthcare worldwide. This infection has rapidly spread across the globe since December 2019, when it was first detected in Wuhan, China.^1^, ^2^ As of December 2021, approximately 275 million cases have been confirmed worldwide, with around 5 million related deaths.^3^

SARS‐CoV‐2 infections in Latin America and the Caribbean represent 12% of recent global infections; this region includes eight of the 10 countries with the highest death rate worldwide.^4^ In Mexico, the first case of COVID‐19 was detected on February 28, 2020.^5^ Since then, our country has been significantly affected by the pandemic, with 3,933,000 cases and 297,000 related deaths reported per December 2021.^3^

In patients living with cancer, there is controversial evidence about the impact of cancer on the evolution of COVID‐19. These patients have been previously recognized as a susceptible population because of immunosuppression status and treatment side effects that may contribute to developing severe forms of infection.^6^, ^7^, ^8^ In addition, as in other non‐cancer populations, older age, male sex, and cardio‐metabolic comorbidities have been associated with a higher risk of mortality,^6^, ^9^ while specific oncologic characteristics such as active cancer treatment and type of neoplasia are inconstantly reported with poor outcomes.^6^, ^10^

A detailed epidemiological, symptoms, and cancer stage assessment is necessary to evaluate and manage these patients. Therefore, this study aims to describe the clinical characteristics, outcomes, and 30 days all‐cause mortality of patients with cancer and SARS‐CoV‐2 infection in a Mexican referral cancer center.

## 2 METHODS

We conducted a retrospective analysis of patients with cancer and confirmed SARS‐CoV‐2 infection from April 19 to December 31, 2020, at the Instituto Nacional de Cancerologia (INCan), a 133‐bed referral, teaching hospital for adult patients with cancer, in Mexico City.

In March 2020, INCan increased its surge capacity for COVID‐19; an exclusive closed 16‐bed isolated ward and an 8‐bed intensive care unit for COVID‐19 patients were conditioned, along with an exclusive triage and ambulatory respiratory attention unit.

All patients with solid or hematological malignancies (HM) and positive laboratory‐confirmed SARS‐CoV‐2 test by real‐time polymerase chain reaction (RT‐PCR) from nasopharyngeal swabs were included.

Information about demographic data, clinical characteristics, comorbidities, oncologic history, Eastern Cooperative Oncology Group performance status (ECOG), oncological treatment, lung computed tomography (CT), laboratory data, co‐infections, treatment for COVID‐19, and outcomes were obtained from epidemiological registries, electronic medical records, and the Microbiology Laboratory. RT‐PCR testing was performed at Instituto Nacional de Enfermedades Respiratorias (INER), a reference laboratory for SARS‐CoV‐2 testing. There was no formal sample size determination, and all patients who met the inclusion criteria were recruited.

Ethics approval was obtained from the Institutional Board Review (Rev/0016/20). Written informed consent was waived because of the nature of data and the unprecedented nature of the COVID‐19 pandemic. Patient confidentiality was protected, and information was secured.

We followed the Strengthening the Reporting of Observational Studies in Epidemiology (STROBE) statements to report the findings.

### 2.1 Definitions

A case of SARS‐CoV‐2 infection was defined as a patient with cancer and positive RT‐PCR assay from a nasopharyngeal or oropharyngeal swab. COVID‐19 englobes patients with SARS‐CoV‐2 infection and acute respiratory illness.

The type of malignancy was divided into two categories: solid tumors and hematologic neoplasm. According to the clinical stage, patients were classified as having advanced cancer if metastatic disease or cancer recurrence were documented at SARS‐CoV‐2 diagnosis. Hematologic malignancies that are not anatomically staged were considered disseminated at diagnosis. Patients on current treatment were further classified if they received cytotoxic agents, immunotherapy (immune checkpoint inhibitors or immune system modulators), or molecular targeted therapy (angiogenesis inhibitors, proteasome inhibitors, signal transduction inhibitors, or monoclonal antibodies). In the case of patients receiving combined regimens of monoclonal antibodies, such as rituximab plus cytotoxic agents (e.g., R‐CHOP) were classified as part of the cytotoxic chemotherapy group. The functionality was measured through ECOG calculated at the last clinical visit.

The follow‐up time was calculated as the difference between the day of COVID‐19 diagnosis and the patients ‘death or last visit, accordingly. Overall and in‐hospital mortality were calculated. Overall mortality was calculated as the number of deaths in patients with COVID‐19 that occurred for the entire cohort. In‐hospital mortality was calculated as the number of deaths during the hospitalization in patients with COVID‐19 in relation to all patients admitted with cancer and COVID‐19.

Best supportive care (BSC) was defined in this study as the group of patients who, at diagnosis of SARS‐CoV‐2 were receiving only supportive treatment, without anticancer therapy, and upon COVID‐19 diagnosis, were deemed to have an overall survival of less than 3‐months and were not candidates for invasive interventions. For patients in BSC with respiratory distress and medical criteria for invasive mechanical ventilation (IMV), do not resuscitate orders for terminal sedation were provided.

### 2.2 Statistical analysis

We conducted a descriptive analysis; quantitative variables were presented according to distribution as medians and interquartile range (IQR); for qualitative variables, frequencies, and percentages were calculated.

Univariate logistic regression was performed to evaluate risk factors for three main outcomes: hospital admission, IMV, and 30‐day mortality. Variables with a *p* < 0.200 were introduced into a multivariate logistic regression analysis to address confounders. Adjusted odds ratios (aOR) with 95% confidence intervals (95% CI) were used to establish the association. For in‐hospital mortality analysis, we excluded patients under BSC. In the analysis for IMV, patients with do‐not‐resuscitate orders were excluded.

SPSS Statistics 25.0 software was used for statistical analysis and graphics and tables were made in Prism version 9.

## 3 RESULTS

During the study period, a total of 433 patients were diagnosed with SARS‐CoV‐2. Two hundred sixty‐eight (62%) were women, and the median age was 55 years (IQR 44–63).

A high proportion of patients had a history of chronic diseases, 129 (30%), 73 (17%), 28 (7%) had one, two, or more than three comorbidities, respectively. Ninety‐three (21%) had diabetes mellitus (DM); 131 (30%) hypertension, and 135 (31%) obesity. One hundred thirty‐seven (32%) patients had a current or previous smoking history.

Three hundred thirty‐nine (78%) patients presented respiratory symptoms at diagnosis, and 94 (22%) were asymptomatic; 6 (8%) patients of the asymptomatic group progressed to symptomatic infection during the following days. The most common symptoms at the onset of illness were cough (54%), fever (43%), dyspnea (37%), and headache (32%).

In 313 (72%) patients, a thoracic CT was performed. The most prevalent findings were ground‐glass infiltrates in 257 (82%) patients, followed by consolidation in 120 (38%), nodules in 113 (36%), and pleural effusion in 42 (13%). One hundred seventy‐six (56%) had bilateral affection.

### 3.1 Type of cancer and chemotherapy

Three hundred forty‐one (79%) had a diagnosis of solid neoplasm, 82 (19%) HM, and 10 (2%) patients were under study for diagnosis and cancer staging. The most common neoplasms were breast (*n* = 81, 19%); lymphoma (*n* = 52, 12%); cervical (*n* = 34, 7%), and colorectal (*n* = 31, 7%). For the stage of cancer, 170 (39%) had advanced cancer. HM were associated with symptomatic SARS‐CoV‐2 infection (OR: 5.27, C.I. 95%; 2.06–13.45, *p* = 0.01).

One hundred thirty‐four patients (31%) were receiving systemic therapy at COVID‐19 diagnosis; 109 (25%) with cytotoxic chemotherapy, and 25 (6%) with immunotherapy or targeted therapy.

ECOG *status* of 0–1, 2, and 3–4 were observed in 271 (63%), 105 (24%), and 54 (12%) patients, respectively. ECOG information was not available in 3 (1%) patients.

### 3.2 Outcomes and secondary infections

Secondary bacterial pneumonia, abdominal sepsis, and bacteremia or invasive fungal infections were documented in 36 (8.3%) patients. The most frequent microorganisms were: *E. coli* (17%), *Pseudomonas* spp. (14%), and *Stenotrophomonas* spp. (14%). Fifteen (3%) patients were treated with amphotericin or voriconazole because of high clinical or radiological suspicion of aspergillosis; only two (13%) were confirmed by positive culture.

**FIGURE 1:**
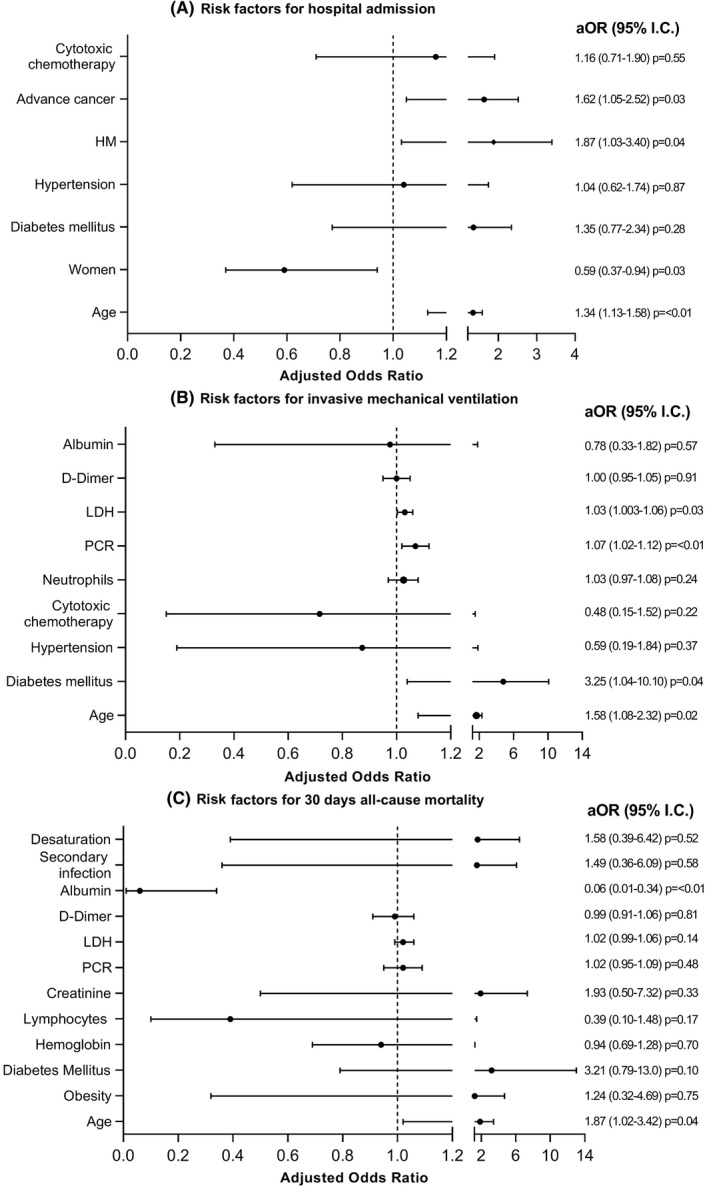
(A, B, C) Multivariate analysis of risk factor associated for hospital admission (A), invasive mechanical ventilation (B), and mortality (C) among patients with cancer and SARS‐CoV‐2. *Notes:* Age was categorized into 10‐year intervals. Desaturation; SpO_2_ <90% in pulsed oximetry. aOR, adjusted odds ratio; HM, hematological malignancies; LDH, lactate dehydrogenase. PCR, polymerase chain reaction; SARS‐CoV‐2, severe acute respiratory syndrome coronavirus 2

Univariate analysis of laboratory findings, demographic, and clinical characteristics for hospitalization, IMV, and mortality are shown in Tables 1, 2, 3, respectively. Two hundred (46%) patients required hospitalization. Variables associated to hospitalization were: age, per 10 years increase (aOR; 1.34, 95% CI; 1.13–1.58, *p* < 0.01); women (aOR; 0.59, 95% CI; 0.37–0.94, *p* = 0.03); hematologic malignancy (aOR; 1.87, 95% CI; 1.03–3.40, *p* = 0.04) and advanced cancer (aOR; 1.62, 95% CI; 1.05–2.52, *p* = 0.03) (Figure 1A).

**TABLE 1:** Univariate analysis of risk factors for hospitalization among patients with cancer and SARS‐CoV‐2 infection (*N* = 433)

Variable	Not hospitalized, *N* = 233 (%)	Hospitalized, *N* = 200 (%)	cOR (CI 95%)	*p* value
Age, median (IQR)^a^	52 (41–61)	57 (48–67)	1.31 (1.14–1.50)	<0.01
Sex				
Women	154 (67)	114 (57)	0.65 (0.44–0.97)	0.03
Smoking	70 (30)	67 (34)	1.17 (0.78–1.76)	0.44
Comorbidities				
Diabetes mellitus	42 (18)	51 (26)	1.56 (0.98–2.47)	0.06
Hypertension	64 (27)	67 (34)	1.33 (0.88–2.00)	0.17
Obesity (BMI ≥30)	74 (32)	61 (31)	0.94 (0.63–1.42)	0.78
Cancer type (*n* = 423)				
Hematologic malignancy	31 (13)	51 (26)	2.25 (1.37–3.70)	<0.01
Stage of cancer (*n* = 302)				
Advanced disease	77 (33)	93 (47)	1.83 (1.22–2.75)	0.03
Chemotherapy 30 days				
No chemotherapy	168 (72)	131 (66)	1	
Cytotoxic chemotherapy	52 (22)	57 (29)	1.41 (0.90–2.18)	0.13
Immunotherapy or targeted therapy	13 (6)	12 (6)	1.18 (0.52–2.68)	0.69

Abbreviations: BMI, body mass index. cOR, crude odds ratio; IQR, interquartile range; SARS‐CoV‐2, severe acute respiratory syndrome coronavirus 2.

^a^ Age was categorized into 10‐years intervals.

**TABLE 2:** Univariate analysis of risk factor for IMV among hospitalized patients with COVID‐19 (*N* = 161)

Variable	Non‐IMV *N* = 116	IMV *N* = 45	cOR (CI 95%)	*p* value
Age, median (IQR)^a^	54 (46–65)	61 (53–66)	1.37 (1.06–1.77)	0.02
Sex				
Women	70 (60)	23 (51)	0.68 (0.34–1.37)	0.29
Smoking	36 (31)	16 (36)	1.23 (0.59–2.53)	0.58
Comorbidities				
Diabetes mellitus	25 (22)	17 (38)	2.21 (1.04–4.66)	0.04
Hypertension	36 (31)	20 (44)	1.78 (0.87–3.60)	0.11
Obesity (BMI ≥30)	37 (32)	16 (36)	1.18 (0.57–2.43)	0.66
Cancer				
Hematological neoplasm (*n* = 157)	30 (26)	15 (33)	1.37 (0.64–2.88)	0.41
Chemotherapy 30 days				
No chemotherapy	75 (65)	36 (80)	1	
Cytotoxic chemotherapy	34 (29)	7 (16)	0.43 (0.17–1.06)	0.07
Immunotherapy or targeted therapy	7 (6)	2 (4)	0.60 (0.11–3.01)	0.53
Stage of cancer (*n* = 142)				
Advanced disease	50 (43)	17 (38)	0.77 (0.36–1.60)	0.48
Laboratory data				
Hemoglobin mg/dl (*n* = 159)	12.4 (9.9–14)	13.0 (10.7–14)	1.03 (0.90–1.17)	0.67
Platelets ×10^3^/μl (*n* = 159)	198 (139–299)	177 (122–290)	0.99 (0.99–1.001)	0.27
Neutrophils ×10^3^/μl (*n* = 159)	4.0 (2.5–7.4)	6.4 (3.4–11.0)	1.07 (1.002–1.14)	0.05
Lymphocytes ×10^3^/μl (*n* = 159)	0.7 (0.5–1.1)	0.7 (0.4–1.1)	1.16 (0.88–1.52)	0.27
Serum creatinine mg/dl (*n* = 157)	0.7 (0.5–0.9)	0.8 (0.6–1.2)	1.28 (0.79–2.05)	0.31
C‐reactive protein mg/dl (*n* = 129)	9.8 (4.9–19.9)	21.6 (12.4–27)	1.06 (1.02–1.09)	<0.01
Ferritin μg/dl (*n* = 80)	77.7 (38.1–178.4)	93.0 (43.1–137.5)	0.99 (0.99–1.003)	0.71
LDH μmol/(h·ml) (*n* = 154)	16.4 (13.4–10.4)	23.1 (18.5–36.6)	1.04 (1.008–1.07)	0.01
D‐Dimer mg/L (*n* = 141)	1.1 (0.7–2)	1.7 (0.9–7.5)	1.07 (1.008–1.13)	0.03
Fibrinogen g/L (*n* = 121)	4.9 (4.0–6.0)	5.5 (4.3–6.5)	1.03 (0.92–1.16)	0.58
Albumin g/dl (*n* = 154)	3.1 (2.7–3.6)	2.9 (2.4–3.4)	0.47 (0.27–0.83)	<0.01

Abbreviations: BMI, body mass index; cOR, crude odds ratio; COVID‐19, coronavirus disease; IMV, invasive mechanical ventilation; IQR, interquartile range; LDH, lactate dehydrogenase.

^a^ Age was categorized into 10‐years intervals. For laboratory values median and interquartile range are reported.

**TABLE 3:** Univariate analysis of risk factor for 30 days all‐cause mortality among hospitalized patients with COVID‐19 (*N* = 172)

Variable	Alive *N* = 131 (%)	Dead *N* = 41 (%)	cOR (CI 95%)	*p*‐value
Age, median (IQR)^a^	55 (46–64)	64 (53–70)	1.51 (1.14–2.00)	<0.01
Sex				
Women	72 (55)	25 (61)	1.28 (0.62–2.61)	0.50
Smoking	43 (33)	13 (32)	0.95 (0.44–2.01)	0.89
Comorbidities				
Diabetes mellitus	30 (23)	14 (34)	1.75 (0.81–3.74)	0.15
Hypertension	44 (34)	13 (32)	0.92 (0.43–1.94)	0.82
Obesity (BMI ≥30)	46 (35)	8 (20)	0.45 (0.19–1.05)	0.07
Cancer type (*n* = 168)				
Hematologic malignancy	36 (27)	10 (24)	0.89 (0.39–2.01)	0.78
Chemotherapy 30 days				
No chemotherapy	86 (66)	29 (71)	1	
Cytotoxic chemotherapy	35 (27)	11 (27)	0.93 (0.42–2.06)	0.86
Immunotherapy or targeted therapy	10 (8)	1 (2)	0.30 (0.03–2.41)	0.26
Stage of cancer (*n* = 153)				
Advanced disease	58 (44)	18 (44)	0.88 (0.42–1.84)	0.74
Laboratory data				
Hemoglobin mg/dl (*n* = 170)	13.0 (11.0–14.1)	10.3 (9.3–13.2)	0.78 (0.67–0.90)	<0.01
Platelets ×10^3^/μl (*n* = 170)	204 (149–299)	153 (80.5–253.5)	1.00 (0.99–1.00)	0.76
Neutrophils ×10^3^/μl (*n* = 170)	4.4 (2.8–7.5)	6.4 (2.5–11.4)	1.01 (0.96–1.06)	0.62
Lymphocytes ×10^3^/μl (*n* = 170)	0.8 (0.5–1.1)	0.6 (0.3–0.85)	0.61 (0.31–1.19)	0.15
Serum creatinine mg/dl (IQR) (*n* = 168)	0.7 (0.5–0.9)	0.9 (0.5–1.8)	2.28 (1.35–3.85)	<0.01
C‐reactive protein mg/dl (*n* = 137)	12.8 (5.8–22.5)	21.0 (11.0–26.9)	1.05 (1.01–1.08)	0.01
Ferritin μg/dl (*n* = 85)	67.5 (38.4–174.8)	111.7 (59.6–259.5)	1.002 (0.99–1.00)	0.16
LDH μmol/(h·ml) (*n* = 163)	17.1 (13.9–22.6)	23.0 (15.7–37.3)	1.03 (1.004–1.05)	0.02
D‐dimer mg/L (*n* = 149)	1.2 (0.7–2.0)	2.4 (1.1–14.2)	1.10 (1.03–1.16)	<0.01
Fibrinogen g/L (*n* = 126)	5.0 (4.1–6.1)	4.7 (4.0–6.0)	0.93 (0.73–1.17)	0.52
Albumin g/dl (*n* = 163)	3.2 (2.8–3.6)	2.3 (2.0–3.0)	0.08 (0.03–0.19)	<0.01
Bacterial or fungal secondary infection	19 (15)	16 (39)	3.77 (1.70–8.34)	<0.01
Steroids therapy	82 (63)	22 (54)	0.69 (0.34–1.40)	0.31
Pulse oximetry (SpO_2_ <90%) (*n* = 169)	66 (50)	28 (68)	2.23 (1.04–4.75)	0.04

Abbreviations: BMI, body mass index; cOR, crude odds ratio; COVID‐19, coronavirus disease; IQR, interquartile range; SpO_2_, peripheral oxygen saturation.

^a^ Age was categorized into 10‐years intervals. For laboratory values median and interquartile range are reported.

At diagnosis 135 (35%) patients had pulsed oximetry <90% and 45 (10%) required IMV during hospitalization. Variables associated to IMV were age per 10 years increase (aOR; 1.58, 95% CI; 1.08–2.32, *p* = 0.02); DM (aOR; 3.25, 95% CI; 1.04–10.10, *p* = 0.04); C‐reactive protein (mg/dl) (aOR; 1.07, 95% CI; 1.02–1.12, *p* < 0.01) and lactate dehydrogenase (LDH) (μmol/(h ml) [aOR; 1.03, 95% CI; 1.003–1.06, *p* = 0.03]) (Figure 1B).

Mortality within 30 days of diagnosis was documented in 76 (18%) patients: 29 (38%) were under terminal sedation. Sixty‐nine (35%) patients died during the hospital stay. Risk factors related with mortality were age, per 10 years increase (aOR; 1.87, 95% CI; 1.02–3.42, *p* = 0.04) and hypoalbuminemia (aOR; 0.06, 95% CI; 0.01–0.34, *p* < 0.01) (Figure 1C).

We did not find differences in the proportion of patients admitted to the hospital (49% vs. 43%, *p* = 0.17) and overall, 30‐day mortality (18% vs. 17%, *p* = 0.95) when comparing the first and second half of 2020 (April–July vs. August–December).

## 4 DISCUSSION

This cohort of patients with cancer and COVID‐19 is one of the few reported in Latin America and the first in our country to describe clinical characteristics and outcomes. Only one study in Mexico described the prevalence of non‐communicable diseases in patients with COVID‐19; immunosuppressed patients had higher mortality, but no details on cancer type or other oncologic related variables were evaluated.^11^ Mexican population differs from the high‐income countries in many characteristics, which underscores the relevance of describing a cohort more similar to low and middle‐income countries.

In our cohort, the most common malignancies were breast and cervical cancer, which explains why most of the patients affected by COVID‐19 were women. The median age of our patients is almost 15 years younger than other international reports in patients with cancer, such as the COVID‐19 Cancer and Consortium (CCC19), the LEOSS registry, or the UKCCMP cohort, which are multicenter studies including mainly centers from the USA, Canada, the United Kingdom, and Western Europe.^6^, ^9^, ^12^ These differences in age distribution reflect the differences in population age structure between our country and high‐income countries; in the 2020 census, only 3.8% of the Mexican population were ≥ 60 years,^13^ compared to 16% in the US in 2019. In Latin America, a publication of cancer patients with COVID‐19 from Brazil still report an older population than ours, but with a difference of only 5–6 years.^14^

There was a high prevalence of obesity, DM, and hypertension in our patients, consistent with the prevalence described in the non‐cancer population in Mexico^15^; it reflects the multimorbidity characteristics and the burden of disease of our population.

A majority of our patients had solid tumors, similar to other series in patients with cancer and COVID‐19.^6^, ^16^ Thirty‐nine percent of patients had advanced cancer, similar to that reported in the series by Lee et al.^9^ in the UK and the report from Nader‐Manta from Brazil.^17^ However, compared to the results by Kuderer, in which only 11% of patients had active or progressive neoplasia,^6^ the proportion of advanced cancer was much higher.

Regarding the clinical presentation, 22% of our patients were asymptomatic. The latter may be explained by the early initiation of the screening program for patients undergoing elective surgery and, to a lesser extent, detecting suspicious cases through CT or Positron Emission Tomography (PET) studies for unrelated reasons to COVID‐19. The percentage is lower compared to that for the general population (40%).^18^ In the LEOSS cohort, the proportion of asymptomatic patients was 13.5%. It is worth mentioning that asymptomatic individuals in our cohort, for the most part, had not received any cancer treatment in the previous months.^12^

Concerning the clinical presentation of COVID‐19, 35% of our patients presented oxygen saturation less than 90% during their first medical evaluation, the double compared to the LEOSS registry.^12^ Our patients generally arrived late on their disease, with advanced stages of infection when diagnosing COVID‐19. This late presentation is also reported in other Mexican series^19^ and partially explains the high hospitalization rate in our patients, near 50%, that is also higher than other Mexican reports in non‐cancer patients, and information from the Mexican Ministry of Health database.^15^

The 30‐day mortality rate was almost 20%. This mortality rate is twice the overall COVID‐19 related mortality reported in the general Mexican population (9%)^19^ but similar to other publications of patients with cancer, with mortality between 13 and 28%.^6^, ^9^, ^12^ Previous analyses in cancer studies have reported that these patients carry a three times higher risk of severe COVID‐19 than the non‐cancer population; in some reports, the risk is higher in active cancer.^20^

Regarding risk factors related to the different outcomes, DM, age, high C‐reactive protein, and LDH were associated with an increased risk of IMV. Many studies report comorbidities to be associated with severe disease. Type 2 DM has been reported as a risk factor for severe COVID in the general Mexican population.^15^, ^21^ Moreover, undiagnosed DM and prediabetes were associated with worse outcomes from SARS‐CoV‐2 infection in a Mexican cohort of hospitalized patients.^22^ In our analysis, we could not include prediabetes or in‐hospital hyperglycemia as potential risk factors for severe disease. Obesity has also been reported as a risk factor for progressive disease in many cohorts.^15^, ^23^ Likewise, cohorts of Mexican patients without cancer have reported obesity and multimorbidity as risk factors as well. Thirty‐one percent of our patients were overweight or obese, but we could not demonstrate a statistical association between these two variables, nor for hypertension or DM. Interestingly, obesity has not been associated with mortality in other cancer and COVID‐19 cohorts.^6^ Some undetected effect modification could affect the association between obesity and COVID‐19 related outcomes. We did not conduct any interaction test.

Regarding age, there was a strong association of older age with hospitalization and good evidence of an association with IMV and mortality. Age has been reported as a risk factor in many cohorts. In the COVID‐19 Cancer and Consortium (CCC19), factors associated with mortality were age, male sex, number of comorbidities, and active cancer.^6^ The UK Coronavirus Cancer Monitoring Project (UKCCMP) reported an association with male sex, advanced age, and comorbidities.^9^ All these cohorts report a population older than ours, so it is interesting to find that this association persists even in a younger cohort.

Hypoalbuminemia has been associated with critically ill patients and mortality in several studies.^24^ It has also been described as a risk factor in patients with HM and influenza pneumonia^25^ and, recently, in severe COVID‐19.^26^ It is difficult to establish if decreased albumin is part of the pathogenesis of severe COVID‐19, or in part, reflects these patients' chronic and more advanced stage of disease. Low albumin levels can potentially help to identify patients at an increased risk of severe disease and assist clinicians in the process of decision making.

We did not find an association between cytotoxic chemotherapy or targeted therapy with the outcomes measured. The UKCCMP also explored previous cancer therapies as a risk of severe COVID, but neither cytotoxic chemotherapy in the last 4 weeks nor targeted therapy, immunotherapy, or radiotherapy were associated with mortality.^9^ In the CCC19 cohort, there was no association between any cancer therapy and mortality. As described in other cohorts, it would seem that patients with cancer carry a higher risk of severe COVID‐19 and/or mortality beyond chemotherapy or the cancer status.^6^ Hematologic neoplasms and advanced cancer stage have also been described to increase the hospitalization rate, but none correlate with a higher mortality risk.

Regarding COVID‐19 treatment, access to COVID‐directed treatment in Mexican institutions has been mainly concentrated in research protocols or COVID‐reconverted hospitals, and most COVID‐19 treatments have relied on steroids. Steroids have shown an impact on mortality in patients with COVID‐19 with oxygen requirements.^27^ In our institution, steroids were part of the standard of care in hospitalized patients with oxygen requirements from July 2020. Therefore, after July 2020, the majority of hospitalized patients received steroids. Before that date, patients who were put on steroids were usually on a case‐by‐case analysis and used to be the ones with a more severe presentation of COVID‐19 and were not initiated early at that time. This probably explains why steroids had no impact on mortality in the multivariate analysis.

This is the first study describing a large cohort of cancer and COVID‐19 throughout the COVID‐19 spectrum and different outcomes in Mexico, and one of the few in Latin America. We report a relatively young cohort of patients with advanced cancer, predominantly female, reflecting the epidemiology of cancer in our country and many other countries in the region. Mortality was similar to that reported in high‐income countries, with age and low albumin as the main factors associated with 30‐day mortality. Cancer‐related factors were not found to be associated with a worse prognosis.

### 4.1 Limitations

This study has some limitations. It is a retrospective study with data collected as the pandemic was ongoing, and we cannot ascertain the causality of all the associations found. Data reported here only represents one center, so the results might not be generalizable to the whole population. However, the INCan is a referral cancer center receiving patients from many country regions. Also, we did not look for specific interaction effects that could have affected the interpretation of some of the associations.

## CONFLICT OF INTEREST

No conflict of interests was declared by the authors.

## AUTHOR CONTRIBUTIONS

Daniel de‐la‐Rosa‐Martinez: SCD, DA, AI, ME, FA. Mercedes Aranda‐Audelo: SCD, DA, AI, ME, FA. Alexandra Martin‐Onraet: SCD, DA, AI, ME, FA. Beda Islas‐Muñoz: DA, ME, FA. Carolina Perez‐Jimenez: DA, ME, FA. Pamela Alatorre‐Fernandez: DA, ME, FA. Patricia Cornejo‐Juarez: SCD, DA, AI, ME, FA. Erika Ruiz‐Garcia: SCD, DA, ME, AI. PA. Paola Zinser‐Peniche: DA, AI, FA. Luis Ortiz‐Luna: DA, AI, FA. Abelardo Meneses‐Garcia: ME, AI, FA. Angel Herrera‐Gomez: AI, ME, FA. Diana Vilar‐Compte: SCD, DA, AI, ME, FA, PA.

## ETHICS STATEMENT

The study was approved by the institutional review board (Rev/0016/20). Written informed consent was waived because of the nature of data and the unprecedented nature of the COVID‐19 pandemic. Patient confidentiality was protected, and information was secured.

## Data Availability

Data available on request due to privacy/ethical restrictions.
